# Genetic Diversity and Pathogenic Features in Klebsiella pneumoniae Isolates from Patients with Pyogenic Liver Abscess and Pneumonia

**DOI:** 10.1128/spectrum.02646-21

**Published:** 2022-03-30

**Authors:** Lin Gan, Chao Yan, Jinghua Cui, Guanhua Xue, Hanyu Fu, Bing Du, Hanqing Zhao, Junxia Feng, Yanling Feng, Zheng Fan, Pan Mao, Tongtong Fu, Ziying Xu, Shuheng Du, Shiyu Liu, Rui Zhang, Qun Zhang, Nannan Li, Xiaohu Cui, Xiaoran Li, Yao Zhou, Lei Huang, Jing Yuan

**Affiliations:** a Department of Bacteriology, Capital Institute of Pediatrics, Beijing, China; b State Key Laboratory of Infectious Disease Prevention and Control, National Institute for Communicable Disease Control and Prevention, Collaborative Innovation Center for Disease Control and Prevention, Beijing, China Center for Diagnosis and Treatment of Infectious Diseases, Chinese Center for Disease Control and Prevention, Beijing, China; c Treatment and Research Center for Infectious Diseases, The Fifth Medical Center of PLA General Hospital, Beijing, China; National University Hospital

**Keywords:** *Klebsiella pneumoniae*, pyogenic liver abscess, pneumonia, virulence, antibiotic resistance

## Abstract

While Klebsiella pneumoniae is a common cause of nosocomial and community-acquired infections, including pneumonia and pyogenic liver abscess, little is known about the population structure of this bacterium. In this study, we investigated the prevalence and molecular characteristics of K. pneumoniae isolates from carriers, pyogenic liver abscess patients, and pneumonia patients, and genomic and phenotypic assays were used to determine the differences among the isolates. A total of 232 K. pneumoniae isolates were subtyped into 74 sequence types (STs). The isolates from different sources had their own STs, and the predominant subtypes in liver abscess and pneumonia patients were ST23 and ST11, respectively. Pangenome analysis also distinguished three phylogroups that were consistent with the isolate sources. The isolates collected from liver abscess patients carried significantly more virulence factors, and those from pneumonia patients harbored significantly more resistance genes and replicons. Almost all isolate STs (93/97 [95.88%]) from liver abscesses strongly correlated with the virulence factor salmochelin, while most pneumonia isolate STs (52/53 [98.11%]) from pneumonia did not correlate with salmochelin. The isolates collected from liver abscesses showed higher virulence in the cytotoxicity and mouse models. These data provide genomic support for the proposal that isolates collected from carriers, liver abscess patients, and pneumonia patients have distinct genomic features. Isolates from the different sources are largely nonoverlapping, suggesting that different patients may be infected via different sources. Further studies on the pathogenic mechanisms of salmochelin and other virulence factors will be required.

**IMPORTANCE** While Klebsiella pneumoniae is a common cause of nosocomial and community-acquired infections, including pneumonia and pyogenic liver abscess, little is known about the population structure of this bacterium. We collected 232 isolates from carriers, pyogenic liver abscess patients, and pneumonia patients, and the isolates from different sources had their own sequence types. Pangenome analysis also distinguished three phylogroups that were consistent with the isolate sources. The isolates collected from liver abscess patients carried significantly more virulence factors, and those from pneumonia patients harbored significantly more resistance genes and replicons. Besides, there was a strong link between salmochelin and liver abscess. The isolates collected from liver abscesses also showed higher virulence in the cytotoxicity and mouse models. Isolates collected from different sources have distinct genomic features, suggesting that different patients may be infected via different sources.

## INTRODUCTION

The Gram-negative bacterium Klebsiella pneumoniae belongs to the family *Enterobacteriaceae*. While the bacterium is widely distributed in host-associated and environmental niches, K. pneumoniae is also a well-known opportunistic pathogen and common causative agent of nosocomial infections (1). K. pneumoniae can cause a series of invasive diseases, such as pneumonia, pyogenic liver abscess, urinary tract infection, endophthalmitis, and meningitis (2–4). It is the most common pathogen of community-acquired pneumonia and hospital-acquired pneumonia. Besides, after K. pneumoniae-caused pyogenic liver abscess was first reported in Taiwan, China, in the 1980s, cases subsequently emerged worldwide, especially in Asia (5, 6).

Some chromosome- and plasmid-encoded features have been described as the causative factors of enhanced Klebsiella virulence. Iron is involved in many crucial bacterial cellular processes, and the chromosome-encoded yersiniabactin and the plasmid-encoded aerobactin and salmochelin can help with iron acquisition. Hypervirulent K. pneumoniae (hv*Kp*) isolates were reported to carry more siderophores compared with classical K. pneumoniae (c*Kp*) isolates, and the ferric uptake system (*kfuABC*) is also more prevalent in hv*Kp* isolates (7–9). Moreover, the bacterial capsule is an identifier of the hypermucoviscous phenotype, which contributes to the inhibition of phagocytosis and antimicrobial peptides and the complementation and induction of host inflammatory responses, and capsule production is regulated by the plasmid-carried genes *rmpA* and *rmpA2* (10–12). In addition, lipopolysaccharide (LPS) is also a critical virulence factor that can trigger the immune response. As a nitrogen source of K. pneumoniae, allantoin is critical for enhanced virulence. An analysis of hv*Kp* isolates found that allantoin metabolism may play an important role in K. pneumoniae liver infection (13). Besides, colibactin has the ability to damage DNA, disrupt the cell cycle, and promote bacterial colonization (14).

Under antibiotic selective pressure and constant evolution, multidrug-resistant (MDR) or even extremely-drug-resistant K. pneumoniae isolates that exhibit resistance to almost all available antibiotics have been frequently reported (15). The extended-spectrum β-lactam (ESBL)-producing and carbapenem-resistant K. pneumoniae isolates have been classified as a critical public health threat by the World Health Organization, and the MDR rates of K. pneumoniae are rising sharply year by year (16, 17). According to a multicenter monitoring scheme that covered 14 provinces of China, K. pneumoniae accounted for 73.9% of 664 carbapenem-resistant *Enterobacteriaceae* (CRE) in clinical samples (18).

K. pneumoniae infection has placed a heavy burden on society. However, there has been a lack of systematic studies, which limits our understanding of the prevalence and features of K. pneumoniae from different sources. In this study, we first collected K. pneumoniae isolates from clinical patients for genotyping to study the epidemiology and genomic characteristics of K. pneumoniae. Interestingly, we found great differences in sequence types (STs) between pyogenic liver abscess patients, pneumonia patients, and carriers, and some STs were only found in one category of patient. Furthermore, comparative genomic and phenotypic assays were used to analyze the commonalities and differences between K. pneumoniae isolates from pyogenic liver abscess patients, pneumonia patients, and carriers.

## RESULTS

### Prevalence and molecular subtyping of K. pneumoniae isolates.

**FIG 1:**
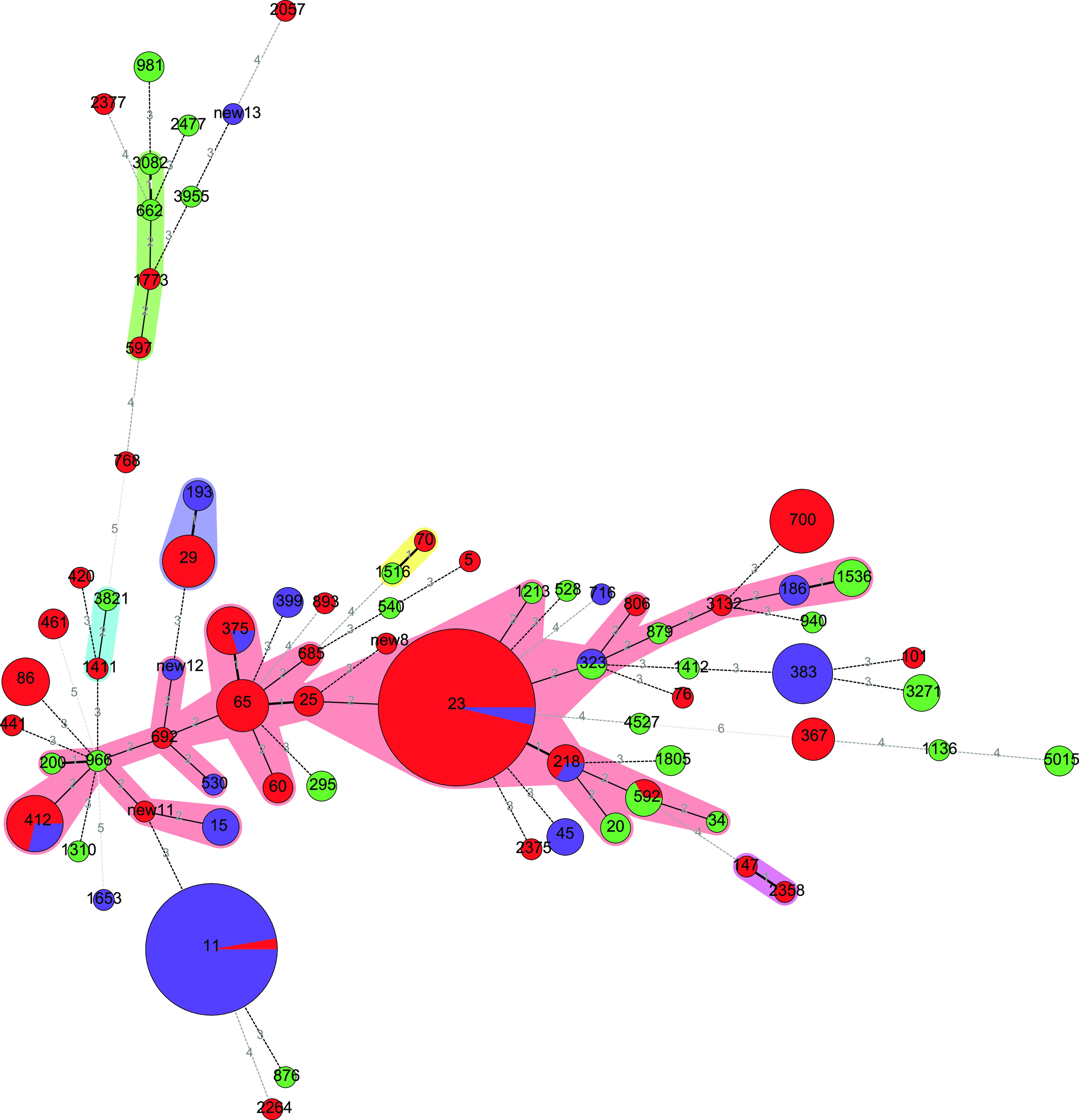
Minimum-spanning tree of K. pneumoniae isolates based on sequence type distribution. Each circle denotes a sequence type (ST), and the colors within the circles represent the isolate source. The size of the circles represents the isolate count, and the number between two circles denotes the number of different housekeeping genes. Red circle denotes the pyogenic liver abscess-sourced isolates, purple circle denotes the pneumoniae-sources isolates, and the green circle denote the carrier-sourced isolates.

The K. pneumoniae isolates were collected from the Anhui, Beijing, Fujian, Henan, Jiangsu, Jiangxi, Shandong, Shanxi, and Zhejiang provinces of China. These 232 isolates were subtyped into 74 STs (Table 1 and Fig. 1). The isolates from carriers were composed of 28 STs. Those from pyogenic liver abscess patients were represented by 37 STs, and the predominant subtype was ST23 (41.94%). The isolates from pneumonia patients included 17 STs, and the predominant subtype was ST11 (54.29%). In subtypes that were collected three times or more, ST29, ST65, ST86, ST367, and ST700 only existed in liver abscess patients, and ST15, ST45, and ST383 were only isolated from pneumonia patients. The subtypes ST11, ST23, ST375, and ST412 were collected from both of the clinical patient categories.

**TABLE 1:** Prevalence and subtyping characteristics of K. pneumoniae isolates

Source	No. (%) of isolates			
	Carrier	Pyogenic liver abscess	Pneumonia	Total
ST11^*a*^		1 (0.81)	38 (54.29)	39
ST15^*b*^			3 (4.29)	3
ST20	2 (5.26)			2
ST23^*a*^		52 (41.94)	2 (2.86)	54
ST25^*c*^		2 (1.61)		2
ST29^*c*^		6 (4.84)		6
ST45^*b*^			3 (4.29)	3
ST60^*c*^		2 (1.61)		2
ST65^*c*^		6 (4.84)		6
ST86^*c*^		5 (4.03)		5
ST186^*b*^			2 (2.86)	2
ST193^*b*^			2 (2.86)	2
ST218^*a*^		2 (1.61)	1 (1.43)	3
ST295	2 (5.26)			2
ST323	1 (2.63)		1 (1.43)	2
ST367^*c*^		4 (3.23)		4
ST375^*a*^		4 (3.23)	1 (1.43)	5
ST383^*b*^			8 (11.43)	8
ST399^*b*^			2 (2.86)	2
ST412^*a*^		5 (4.03)	2 (2.86)	7
ST461^*c*^		2 (1.61)		2
ST592	2 (5.26)	1 (0.81)		3
ST700^*c*^		9 (7.26)		9
ST981	2 (5.26)			2
ST1536	3 (7.89)			3
ST1805	2 (5.26)			2
ST3271	3 (7.89)			3
ST5015	2 (5.26)			2
Other STs^*d*^	19 (50.00)	23 (18.55)	5 (7.14)	47

Total	38 (100.00)	124 (100.00)	70 (100.00)	232

a STs collected from both pneumonia and pyogenic liver abscess patients.

*^b^*STs collected from only pneumonia patients.

*^c^*STs collected from only pyogenic liver abscess patients.

d STs collected only one time.

### Genomic relationships of K. pneumoniae isolates.

**FIG 2:**
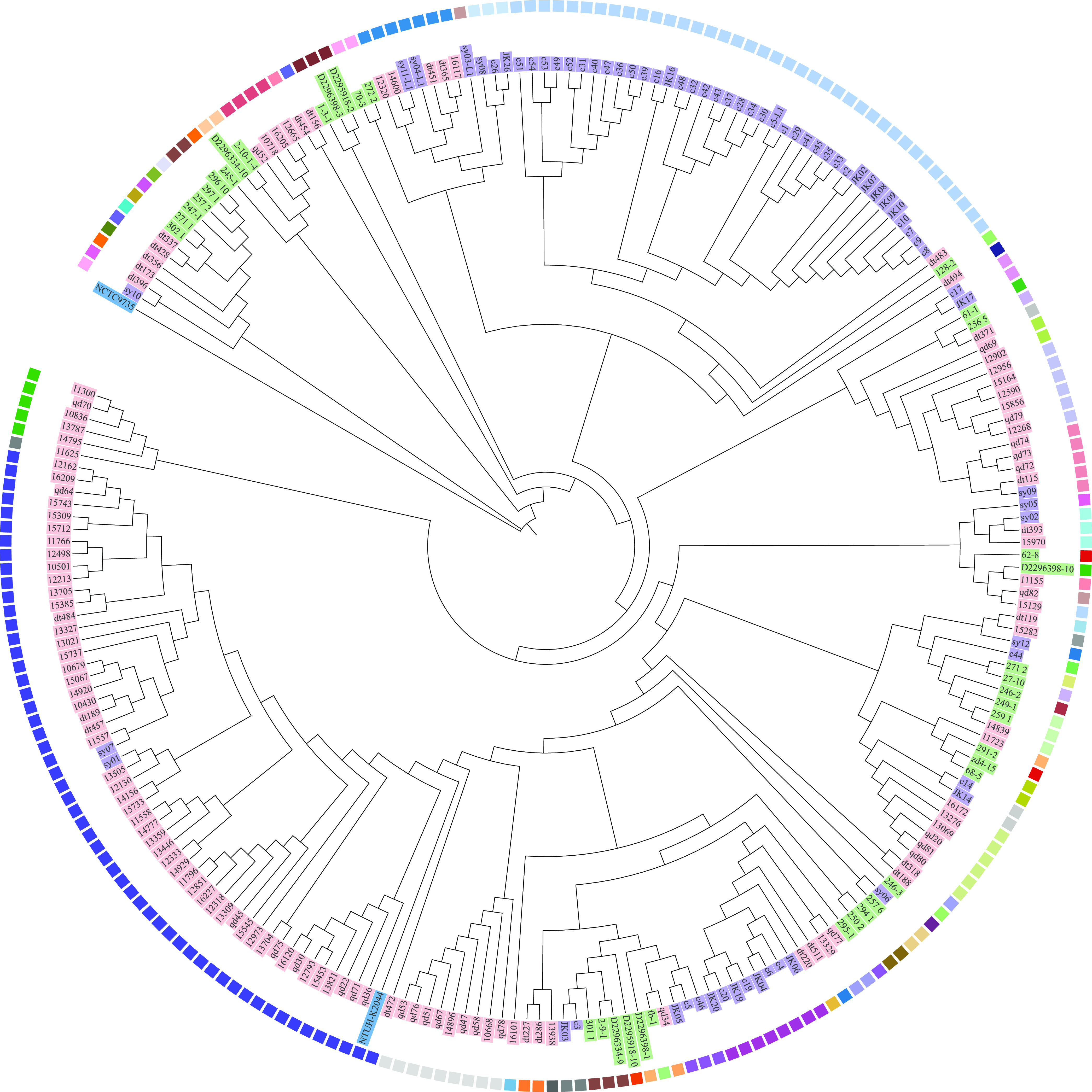
Phylogenetic trees of K. pneumoniae isolates based on core genome SNPs. Phylogenetic tree generated by the neighbor-joining method with 1,000 bootstrap replicates, as implemented in MEGA. Strain NTUH-K2044 was used as a reference and NCTC9735 (K. aerogenes) as an outgroup. Isolates collected from carriers and pyogenic liver abscess and pneumonia patients are shaded with green, pink, and purple label backgrounds, respectively. The squares beside the isolates represent the STs.

The 232 K. pneumoniae isolates were subjected to whole-genome sequencing, and the general genomic characteristics are shown in Table S1 in the supplemental material. Core single-nucleotide polymorphisms (SNPs) were used to construct the phylogenetic tree, and the Klebsiella aerogenes strain NCTC9735 (GCA_900637945.1) was used as an outgroup (Fig. 2). A total of 3,131 core genes were conserved in 234 Klebsiella genomes containing the 232 clinical isolates, NCTC9735, and NTUH-K2044, and we identified 177,953 SNPs within the genes. These K. pneumoniae isolates were divided into several clusters that were consistent with previously defined STs. In general, isolates from liver abscesses and pneumonia patients were placed into relatively distinct clusters, while the isolates from carriers were scattered throughout the phylogenetic tree. Furthermore, no obvious correlation between phylogenetic relationship and isolation site was found.

**FIG 3:**
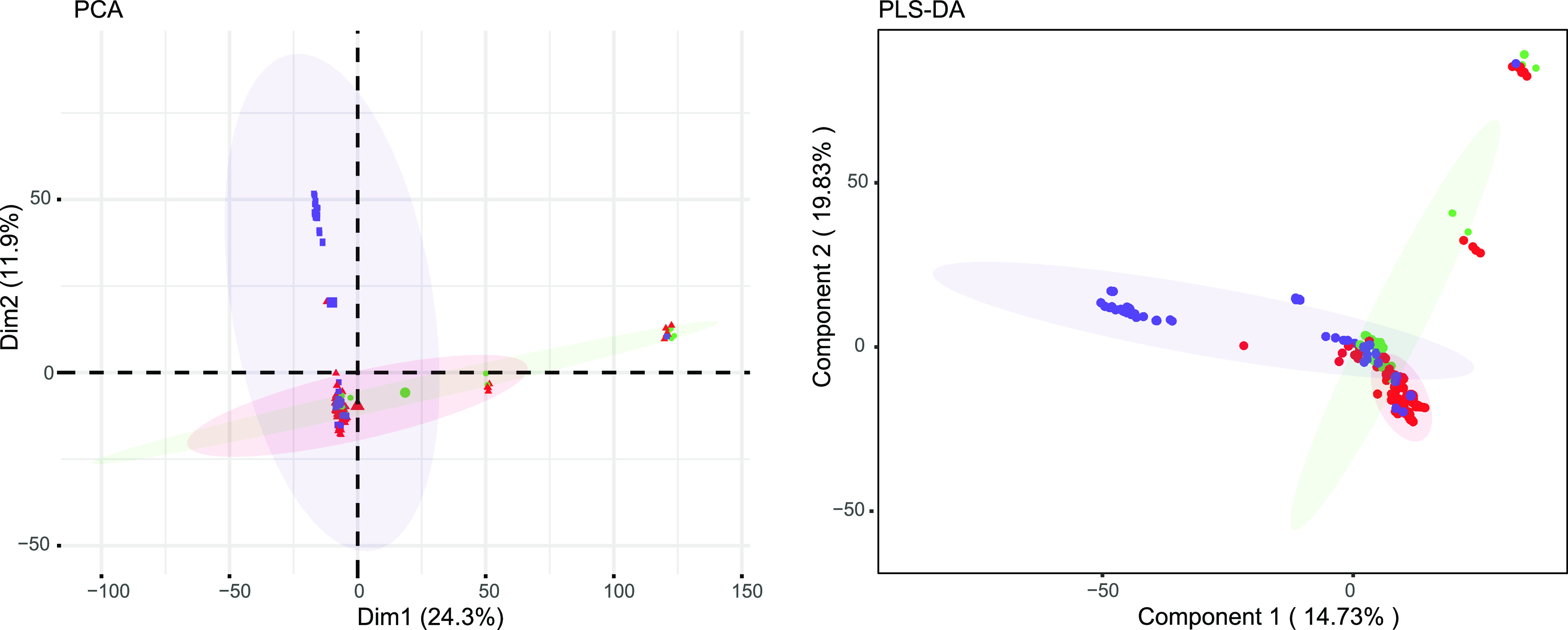
PCA and PLS-DA based on the presence of common (5 to 95% prevalence) accessory genes. Isolates collected from carriers and pyogenic liver abscess and pneumoniae patients are presented by green, red, and purple spots, respectively.

We identified a pangenome of 21,196 accessory genes (including 427 soft genes, 95% ≤ strains < 99%; 2,584 shell genes, 15% ≤ strains < 95%; and 18,185 cloud genes, 0% ≤ strains < 15%) among the Klebsiella genomes, and these genes could be divided into four classes: information storage and processing, cellular processes and signaling, metabolism, and poorly characterized genes (see Fig. S1 in the supplemental material). Furthermore, principal-component analysis (PCA) and partial least-squares discriminant analysis (PLS-DA) (permutational analysis of variance [PERMANOVA], *P < *0.01) of common (prevalence of 5 to 95%) accessory gene contents distinguished three phylogroups that were consistent with the sources of the isolates (Fig. 3). These data provide genomic support for the proposal that isolates collected from carriers, liver abscess patients, and pneumonia patients have distinct genomic features.

### Antimicrobial resistance genes of K. pneumoniae isolates.

Given the importance of resistance genes in the clinic, we performed *in silico* antibiogram analysis of K. pneumoniae isolates within the genomic data sets. Resistance genes associated with the antibiotics aminoglycoside (72 [31.03%]), beta-lactam (231 [99.57%]), fluoroquinolone (212 [91.38%]), fosfomycin (232 [100.00%]), macrolides-lincosamides-streptogramin B (MLS) (23 [9.91%]), phenicol (43 [18.53%]), rifampin (5 [2.16%]), sulfonamide (65 [28.02%]), tetracycline (62 [26.72%]), and trimethoprim (59 [25.43%]) were found in 232 K. pneumoniae isolates (see Fig. S2 and Table S2 in the supplemental material).

**FIG 4:**
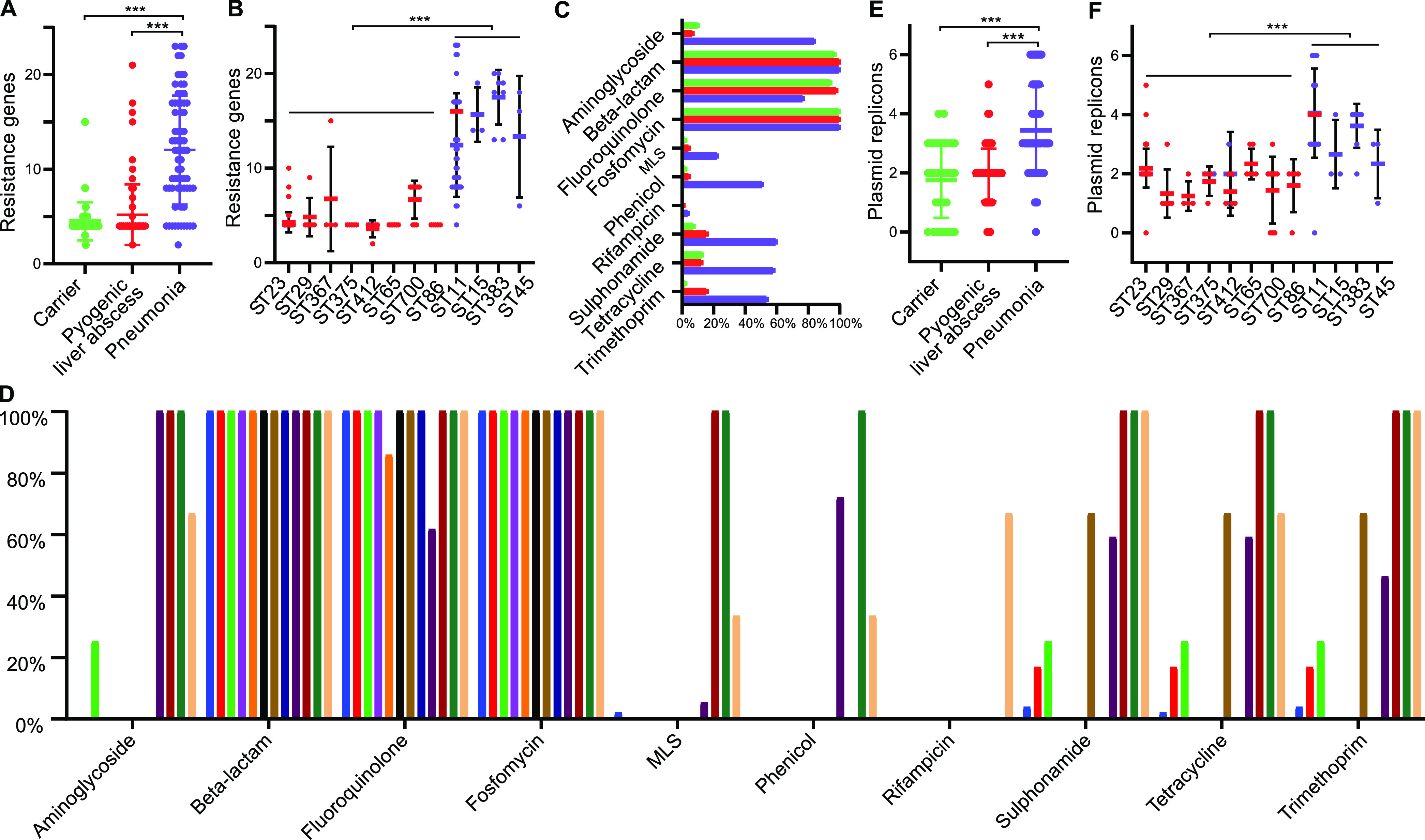
Resistance genes and plasmid replicon analysis of K. pneumoniae isolates. (A and B) Number of resistance genes per isolate for differently sourced isolates (A) and STs (B). (C and D) Frequency of isolates that harbored resistance genes to each antibiotic across differently sourced isolates (C) and STs (D). (E and F) Number of plasmid replicons per isolate in differently sourced isolates (E) and STs (F). In Fig. 4A, 4B, 4C, 4E, and 4F, green, red, and purple spots/ columns are denote the isolates collected from carriers and pyogenic liver abscess and pneumoniae patients, respectively. In Fig. 4D, isolates of ST23, ST29, ST367, ST375, ST412, ST65, ST700, ST86, ST11, ST15, ST383, and ST45 are presented by blue, red, green, purple, orange, black, brown, ultramarine, darkorchid, deepred, loden, and yellow columns, respectively.

One-way analysis of variance (ANOVA) demonstrated that the isolates sourced from pneumonia patients harbored significantly more (*P < *0.01) resistance genes than isolates from carriers and liver abscess patients (Fig. 4A). Isolates of ST11, ST15, ST45, and ST383, which were strongly correlated with pneumonia patients, also contained significantly more resistance genes (one-way ANOVA, *P < *0.01) than the other isolates (Fig. 4B). The same trend was seen in the data for each antibiotic. Almost all isolates harbored beta-lactam- and fosfomycin-associated resistance genes. The isolates collected from pneumonia patients showed significantly more genes (one-way ANOVA, *P < *0.01) that are responsible for resistance to all of the antibiotics mentioned above, except fluoroquinolone, than other clinic isolates (Fig. 4C). In addition, significantly more (one-way ANOVA, *P < *0.01) types of antibiotic-associated resistance genes also existed in isolates of ST11, ST15, ST45, and ST383 (Fig. 4D). The difference between isolates from carriers and liver abscess patients was not statistically significant (one-way ANOVA, *P > *0.05).

### Plasmids of K. pneumoniae isolates.

A total of 212 of the 232 (91.38%) K. pneumoniae isolates carried plasmids. On plasmid typing, replicons IncF (131 [56.47%]), repB (101 [43.53%]), IncH (94 [40.52%]), IncR (62 [26.72%]), Col (57 [24.57%]), IncN (11 [4.74%]), IncL (11 [4.74%]), IncQ (2 [0.86%]), IncC (2 [0.86%]), and IncA (1 [0.43%]) were found in the 212 K. pneumoniae isolates (see Fig. S3 in the supplemental material).

The isolates sourced from pneumonia patients carried significantly more plasmid replicons (one-way ANOVA, *P = *0.000) than isolates collected from carriers and liver abscess patients, and isolates of ST11, ST15, ST45, and ST383 contained significantly more plasmid replicons than other types (one-way ANOVA, *P < *0.01) (Fig. 4E and F). The difference between the isolates from carriers and liver abscess patients was not statistically significant (one-way ANOVA, *P > *0.05).

### Virulence-related gene analysis.

**FIG 5:**
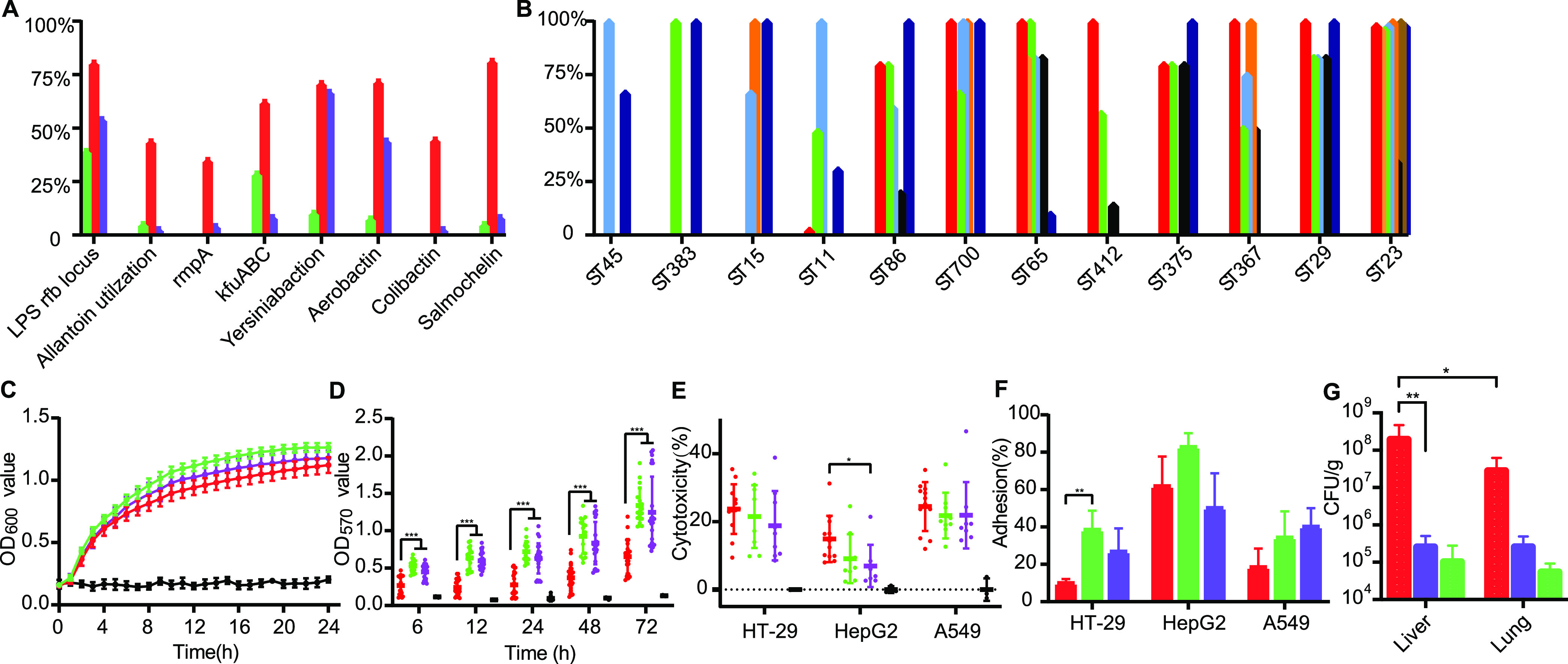
Virulence analysis of K. pneumoniae isolates. (A and B) Frequency of virulence gene clusters among K. pneumoniae isolates from different sources (A) and STs (B). (C to F) Growth curve (C), biofilm formation ability (D), cytotoxicity (E), and cell adhesion rate (F) of K. pneumoniae isolates from different sources. (G) Bacterial loads of K. pneumoniae isolates in the liver and lung. In fig. 5A, 5C, 5D, 5E, 5F, and 5G, green, red, purple and black spots/ columns/ lines are denote the carrier-sourced isolates, pyogenic liver abscess-sourced isolates, pneumoniae-sourced isolates, and PBS, respectively. In Fig. 5B, virulence factors Yersiniabactin, Salmonchelin, Colibactin, Aerobactin, kfuABC, RmpA, Allantoin, and LPS rfb locus are presented by wathet, red, yellow, green, orange, black, brown, and ultramarine columns, respectively.

Genes responsible for virulence factors AcrAB, aerobactin, allantoin, colibactin, enterobactin, KfuABC, the LPS Rfb locus, RcsAB, RmpA, salmochelin, type 3 fimbriae, and yersiniabactin type VI secretion system (T6SS)-I, T6SS-II, and T6SS-III were found in the 232 K. pneumoniae isolates (see Fig. S4 in the supplemental material). There were systematical differences in the distribution of virulence factors among isolates from carriers, pyogenic liver abscess patients, and pneumonia patients, and these differentially distributed factors were salmochelin (109 [46.98%]), colibactin (59 [25.43%]), aerobactin (123 [53.02%]), yersiniabactin (139 [59.91%]), KfuABC (94 [40.52%]), RmpA (46 [19.83%]), allantoin (58 [25.00%]), and the LPS Rfb locus (153 [65.95%]). The isolates collected from liver abscess patients carried significantly more virulence factors (one-way ANOVA, *P < *0.01) than other isolates (Fig. 5A). Isolates of ST23, ST29, ST367, ST375, ST412, ST65, ST700, and ST86, which were strongly correlated with liver abscess patients, also contained significantly more virulence factors (one-way ANOVA, *P < *0.01) than other isolates (Fig. 5B). In addition, there was a strong relationship between salmochelin and isolates sourced from liver abscess patients. All isolates except four from liver abscesses strongly correlated with STs carrying salmochelin, and all isolates except one from pneumonia patients correlated with STs containing no salmochelin (Fig. 5B).

### Virulence of K. pneumoniae *in vitro* and *in vivo*.

We further evaluated the proliferation, biofilm formation, cytotoxicity, cell adhesion, and mouse toxicity of the K. pneumoniae isolates. Proliferation ability was detected by growth curves for culture in LB broth. There were no significantly different growth rates between the isolates from various sources, as shown in Fig. 5C. No significant difference (one-way ANOVA, *P > *0.05) in biofilm-forming ability was found between the isolates from carrier and pneumonia patients, but the isolates collected from liver abscess patients exhibited significantly weaker (one-way ANOVA, *P < *0.001) biofilm-forming ability for 3 to 72 h after incubation (Fig. 5D). The bacterial cytotoxicity and cell adhesion ability were tested in HT-29, HepG2, and A549 cells. Significantly higher cytotoxicity (one-way ANOVA, *P < *0.05) toward HepG2 cells was found for the isolates from liver abscesses, and the isolates from other groups exhibited no significant (one-way ANOVA, *P > *0.05) difference in cytotoxicity (Fig. 5E). No significant differences in HepG2 and A549 cell adhesion (one-way ANOVA, *P > *0.05) were found among isolates from the three sources, but isolates collected from liver abscess patients exhibited a significantly lower (one-way ANOVA, *P < *0.01) adhesion rate in HT-29 cells (Fig. 5F).

The virulence of the K. pneumoniae isolates was also examined *in vivo* using a C57 mouse model of intraperitoneal infection (2 × 10^6^ CFU), from which bacterial loads from the liver and lung were measured 1 day postinfection (dpi). The liver bacterial loads of mice in the liver abscess-sourced isolate-infected group were significantly higher (one-way ANOVA, *P < *0.01) than those of pneumonia-sourced isolate-infected and carrier-sourced isolate-infected mice. In addition, mice infected with the liver abscess-sourced isolates showed significantly higher (one-way ANOVA, *P < *0.05) bacterial loads in the liver than the lung (Fig. 5G).

## DISCUSSION

K. pneumoniae infection is a serious public health threat due to the increasing prevalence of infections caused by MDR K. pneumoniae worldwide (19, 20). According to the most recent statistics from the China Antimicrobial Surveillance Network, K. pneumoniae accounts for 13.86% of clinical bacteria in China. K. pneumoniae has the ability to cause invasive diseases, such as pneumonia and pyogenic liver abscesses. However, little is known about the genetic diversity and structure of K. pneumoniae populations from carriers, pyogenic liver abscesses, and pneumonia patients or how these correlate with the ecology of the bacterium and its capacity to cause various infectious diseases. To address the questions mentioned above, we explored the prevalence, genetic characteristics, and preliminary virulence of K. pneumoniae populations in this study.

ST23 accounted for 41.94% of the 37 STs of isolates from liver abscess patients, and isolates of ST29, ST65, ST86, ST367, and ST700 only existed in liver abscess patients. The prevalences of STs in the pyogenic liver abscess patients were similar to the results from a previous study of a Chinese cohort (21). Of the K. pneumoniae STs previously reported, ST23 has spread globally through multiple international transmissions and is responsible for the majority of pyogenic liver abscess (22, 23). Of the 17 STs of isolates collected from pneumonia patients, the predominant subtype was ST11 (54.29%), and isolates of ST15, ST45, and ST383 were only observed in pneumonia patients. ST11 has been a common subtype in hospital-acquired pneumonia (24, 25). The isolates of ST11, ST23, ST375, and ST412 were collected from both categories of clinical patients. Additionally, no obvious correlation between ST and location or time of isolation from carriers was found, and the distribution of STs was relatively disperse. Similar to the results of molecular typing, core genome and pangenome analyses demonstrated that isolates from carriers and liver abscess and pneumonia patients had individual genomic features, suggesting that different patients may be infected by different sources and evolutionary branches. In addition to being common in the community and hospital, K. pneumoniae can be found in a wide range of host-associated and environmental niches, which provides theoretical support for our hypothesis (19, 26).

Using *in silico* antibiogram analysis, we found that isolates from the pneumonia patients carried more resistance genes than those from carriers and liver abscess patients, and the pneumonia-related STs harbored more resistance genes. Combined with the epidemiological evidence, our findings suggest most pneumonia patient-sourced isolates may be transmitted through hospitals or communities and have a higher probability of the horizontal transfer of antimicrobial resistance genes. The results of the plasmid replicon analysis provided additional evidence for the above interpretation. Pneumonia patient-sourced isolates carried more replicons than those from other sources. Previous studies have demonstrated that multiple-replicon plasmids are more capable of carrying resistance genes than non-multiple-replicon plasmids, which may be a vital mechanism underlying Klebsiella responses to high antibiotic pressure (27, 28). Much of the antimicrobial resistance of K. pneumoniae is a consequence of acquired plasmids, and plasmids such as IncFIIK have been associated with the expression of carbapenemase (29). Further studies will be required to confirm the above inferences.

It is also interesting to note that the isolates from liver abscess patients carried more virulence factors, such as aerobactin, salmochelin, colibactin, allantoin, RmpA, and KfuABC, than those from other sources. In isolates that differentially carried virulence systems or genes, salmochelin was strongly linked to the isolates collected from liver patients. Almost all isolates (93/97 [95.88%]) from liver abscesses strongly correlated with STs carrying salmochelin, and most isolates (52/53 [98.11%]) from pneumonia patients were correlated with STs containing no salmochelin. Salmochelin is a C-glucosylated siderophore encoded by the *iroBCDEN* genes. Glucosylation has been identified to be an evasion mechanism against mammalian immune systems, and the importance of salmochelin in bacterial growth and enhanced virulence has also been verified (30, 31). Although virulence could not be attributed to a single factor, certain intrinsic relationships were noticed in the pyogenic liver abscess-sourced isolates, which will require further investigation. To further explore the virulence differences among the K. pneumoniae isolates, we conducted virulence-related tests. Contrary to expectations, the biofilm formation ability of the liver abscess-sourced isolates was weaker than that of the others, and there was no difference in the growth rate or cell adhesion percentage among the K. pneumoniae isolates. A previous study found that the K. pneumoniae hypermucoviscosity determined by capsule might affect its adhesion and invasion, which could be relevant to our results (32). However, the isolates collected from liver abscesses showed higher virulence in the cytotoxicity and mouse models. Notably, liver abscess-sourced isolate-infected mice showed higher bacterial loads in the liver than the lungs, which was consistent with the epidemiological information on the liver abscess-sourced strains. Further studies into the pathogenic mechanisms of salmochelin and other virulence factors will be required.

Our results provide genomic support for the proposal that K. pneumoniae isolates collected from carrier and liver abscess and pneumonia patients have distinct genomic features. Isolates from different sources were largely nonoverlapping, suggesting that different patients may be infected by different sources and evolutionary branches. Moreover, there was a strong link between salmochelin and the isolates sourced from liver abscess patients, and its pathogenic mechanism requires further investigation.

## MATERIALS AND METHODS

### Bacterial strains.

A total of 232 K. pneumoniae isolates (including 38 isolates from carriers, 124 isolates from pyogenic liver abscess patients, and 70 isolates from pneumonia patients) were collected from nine provinces of China in 2013 to 2020 (Table S1). The K. pneumoniae reference strain NTUH-K2044 (GCA_000009885.1, collected from a pyogenic liver abscess patient), ATCC BAA-2146 (GCA_000349285.2, collected from a pneumonia patient), and 232 isolates were cultured in Luria-Bertani (LB) medium at 37°C.

### Genome sequencing, assembly, annotation, and ST analysis.

For genomic comparison and assessment, 232 K. pneumoniae isolates were selected for whole-genome sequencing (Table S1). Purified DNA was extracted by the Wizard genome DNA purification kit according to the manufacturer’s introduction. Sequencing was performed on the Illumina HiSeq PE150 platform by the Institute of Microbiology, Chinese Academy of Sciences, using K. pneumoniae NTUH-K2044 as the reference strain, and the genome sequences were assembled by SOAP denovo (version 2.04) and Prokka (version 1.14.6).

Multilocus sequence typing (MLST) was used for ST identification. On submitting the sequences of the housekeeping genes *gapA*, *infB*, *mdh*, *pgi*, *phoE*, *rpoB*, and *tonB* to the Institute Pasteur K. pneumoniae MLST database (https://bigsdb.pasteur.fr/klebsiella/), the STs were confirmed (33). A minimum-spanning tree based on the STs of K. pneumoniae from different sources was constructed using BioNumerics 4.0 software and based on 232 K. pneumoniae isolates.

### Core and pangenome analyses.

The core genome single-nucleotide polymorphisms (SNPs) of 232 K. pneumoniae isolates were extracted by Snippy (version 4.4.0) and Gubbins (version 2.4.1), using NTUH-K2044 as the reference strain (34). SNPs not positioned at a recombination region and with a distance between 2 SNP sites of >5 reads were screened for subsequent analysis. The neighbor-joining tree was constructed by MEGA-X based on the core genome SNPs, and bootstraps were performed with 1,000 replicates. Pangenome analysis was applied with Roary (version 3.11.2), and the tool eggNOG-mapper was used for gene functional classification (35, 36). Pangenome-based principal-component analysis (PCA) and partial least-squares discriminant analysis (PLS-DA) were performed in SIMCA (version 14.1) and calculated using permutational multivariate analysis of variance (PERMANOVA) as previously published (7).

### Virulence genes, antimicrobial resistance genes, and plasmid analysis.

For virulence identification, the 232 K. pneumoniae isolates were analyzed on VFanalyzer from the Virulence Factors of Pathogenic Bacteria (http://www.mgc.ac.cn/VFs/main.htm) using NTUH-K2044 as the reference strain (37). The major virulence factors in the database of Klebsiella contain adherence, biofilm formation, efflux pump, immune evasion, iron uptake, nutritional factor, regulation, secretion system, serum resistance, and toxin-related genes (10).

*In silico* antibiograms were predicted by Resfinder (version 4.0). The Resfinder database contains resistance genes associated with the antibiotics aminoglycoside, beta-lactam, colistin, fluoroquinolone, fosfomycin, fusidic acid, glycopeptide, macrolides-lincosamides-streptogramin B (MLS), nitroimidazole, oxazolidinone, phenicol, pseudomonic acid, rifampin, sulfonamide, tetracycline, and trimethoprim. The genes responsible for resistance to these antibiotics are listed on the website https://cge.cbs.dtu.dk/services/ResFinder/database.php (38). The plasmid replicons were detected and typed by PlasmidFinder (version 2.0.1), using the *Enterobacteriaceae* plasmid database; the database contains 116 replicons that were identified in 559 fully sequenced plasmids (39).

### *In vitro* growth and biofilm formation assay.

A total of 31 K. pneumoniae isolates, including 5 isolates from carriers, 17 isolates from pyogenic liver abscess patients (containing NTUH-K2044), and 9 isolates from pneumonia patients (containing ATCC BAA-2146), were selected for the *in vitro* growth assay. In addition, the prevalent STs, such as ST11, ST15, ST23, ST29, ST65, ST86, ST383, ST412, and ST700, were included. The log-phase Klebsiella isolates were transferred to LB broth at a 1:100 dilution and cultured for 24 h, and the values for optical density at 600 nm (OD_600_) were detected with a Bioscreen C microbiology reader each hour.

Eleven representative K. pneumoniae isolates, including four isolates from pyogenic liver abscess patients (containing NTUH-K2044, two isolates of ST23, and one isolate of ST700), four isolates from pneumonia patients (containing ATCC BAA-2146, two isolates of ST11, and one isolate of ST383), and three isolates from carriers were selected for the biofilm formation assay and subsequently virulence assays. The OD_570_ values after crystal violet staining were evaluated at 6, 12, 24, 48, and 72 h postincubation (40).

### Cytotoxicity, cell adhesion assays, and mouse model.

Cytotoxicity assays were used to evaluate cell lactate dehydrogenase (LDH) release, which denotes cell damage. HT-29 (4 × 10^4^), HepG2 (4 × 10^4^), and A549 (8 × 10^4^) cells were incubated in 96-well plates the day before infection with bacteria at a multiplicity of infection (MOI) of 100. The concentration of LDH in the cell supernatants was measured according to the manufacturer’s instructions (Promega, G1780) after 3 h of incubation. HT-29 (2 × 10^5^), HepG2 (2 × 10^5^), and A549 (4 × 10^5^) cells were incubated in 24-well plates the day before adhesion with the log-phase bacteria at an MOI of 100. After 2 h of infection, the bacteria were released from the cells by adding phosphate-buffered saline (PBS) containing 0.1% Triton X-100 and incubated on LB plates for colony counting (41).

For construction of the mouse infection model, we selected 6- to 8-week-old male C57 mice (Charles River). Eleven K. pneumoniae isolates, as mentioned above, representing four pyogenic liver abscess-sourced isolate-infected, four pneumonia-sourced isolate-infected, and three carrier-sourced isolate-infected groups were injected intraperitoneally at 2 × 10^6^ CFU, and the bacterial loads in the liver and lung were counted 1 day postinfection (dpi). Mice were euthanized for analysis, and the experimental procedures mentioned above were approved by the medical ethics committee of the Capital Institute of Pediatrics and carried out by an individual with license no. DWLL2021009.

### Data availability.

Whole-genome sequencing files were submitted to the National Center for Biotechnology Information (https://submit.ncbi.nlm.nih.gov/). For specific genome accession numbers, please see Table S1 in the supplemental material.
