# Supplementary material for: Genetic Diversity and Pathogenic Features in Klebsiella pneumoniae Isolates from Patients with Pyogenic Liver Abscess and Pneumonia
Source: Microbiol Spectr. 2022 Mar 30;10(2):e02646-21. doi: 10.1128/spectrum.02646-21 (PMC9045331; doi:10.1128/spectrum.02646-21)
Supplement: SUPPLEMENTAL FILE 1 — Supplemental material. Download SPECTRUM02646-21_Supp_1_seq6.pdf, PDF file, 2.2 MB [file spectrum02646-21_supp_1_seq6.pdf]

Figure Legends

Figure S1 Heatmap of accessory genes in 232 *K. pneumoniae* isolates.

Squares beside isolates represent the isolation sources and STs. Squares listed on the right represent clusters of orthologous groups (COG).

Figure S2 Heatmap of resistance genes in 232 *K. pneumoniae* isolates.

Squares beside the isolates represent isolation sources and STs. Squares listed on the right represent antibiotic-related resistance genes.

Figure S3 Heatmap of plasmid replicons in 232 *K. pneumoniae* isolates.

Squares beside the isolates represent isolation sources and STs. Squares listed on the right represent plasmid replicons.

Figure S4 Heatmap of virulence genes in 232 *K. pneumoniae* isolates.

Squares beside the isolates represent isolation sources and STs. Squares listed on the right represent virulence genes.

Figure S1

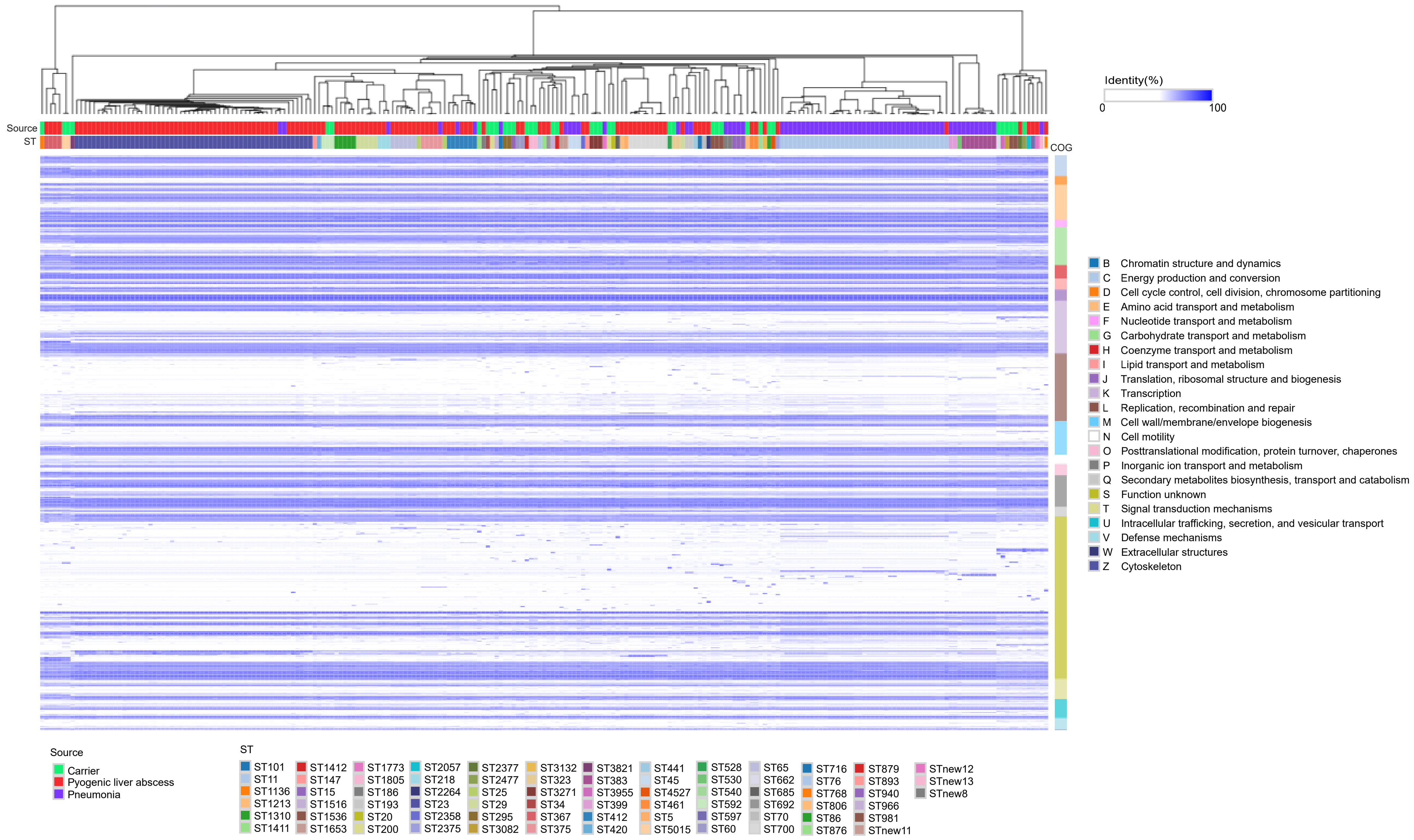
Identity(%)
0
100
Source
ST
COG
B Chromatin structure and dynamics
C Energy production and conversion
D Cell cycle control, cell division, chromosome partitioning
E Amino acid transport and metabolism
F Nucleotide transport and metabolism
G Carbohydrate transport and metabolism
H Coenzyme transport and metabolism
I Lipid transport and metabolism
J Translation, ribosomal structure and biogenesis
K Transcription
L Replication, recombination and repair
M Cell wall/membrane/envelope biogenesis
N Cell motility
O Posttranslational modification, protein turnover, chaperones
P Inorganic ion transport and metabolism
Q Secondary metabolites biosynthesis, transport and catabolism
S Function unknown
T Signal transduction mechanisms
U Intracellular trafficking, secretion, and vesicular transport
V Defense mechanisms
W Extracellular structures
Z Cytoskeleton
Source
Carrier
Pyogenic liver abscess
Pneumonia
ST
ST101 ST11 ST1136 ST1213 ST1310 ST1411
ST1412 ST147 ST15 ST1516 ST1536 ST1653
ST1773 ST1805 ST186 ST193 ST20 ST200
ST2057 ST218 ST2264 ST23 ST2358 ST2375
ST2377 ST2477 ST25 ST29 ST295 ST3082
ST3132 ST323 ST3271 ST34 ST367 ST375
ST3821 ST383 ST3955 ST399 ST412 ST420
ST441 ST45 ST4527 ST461 ST5 ST5015
ST528 ST530 ST540 ST592 ST597 ST60
ST65 ST662 ST685 ST692 ST70 ST700
ST716 ST76 ST768 ST806 ST86 ST876
ST879 ST893 ST940 ST966 ST981 STnew11
STnew12 STnew13 STnew8

Figure S2

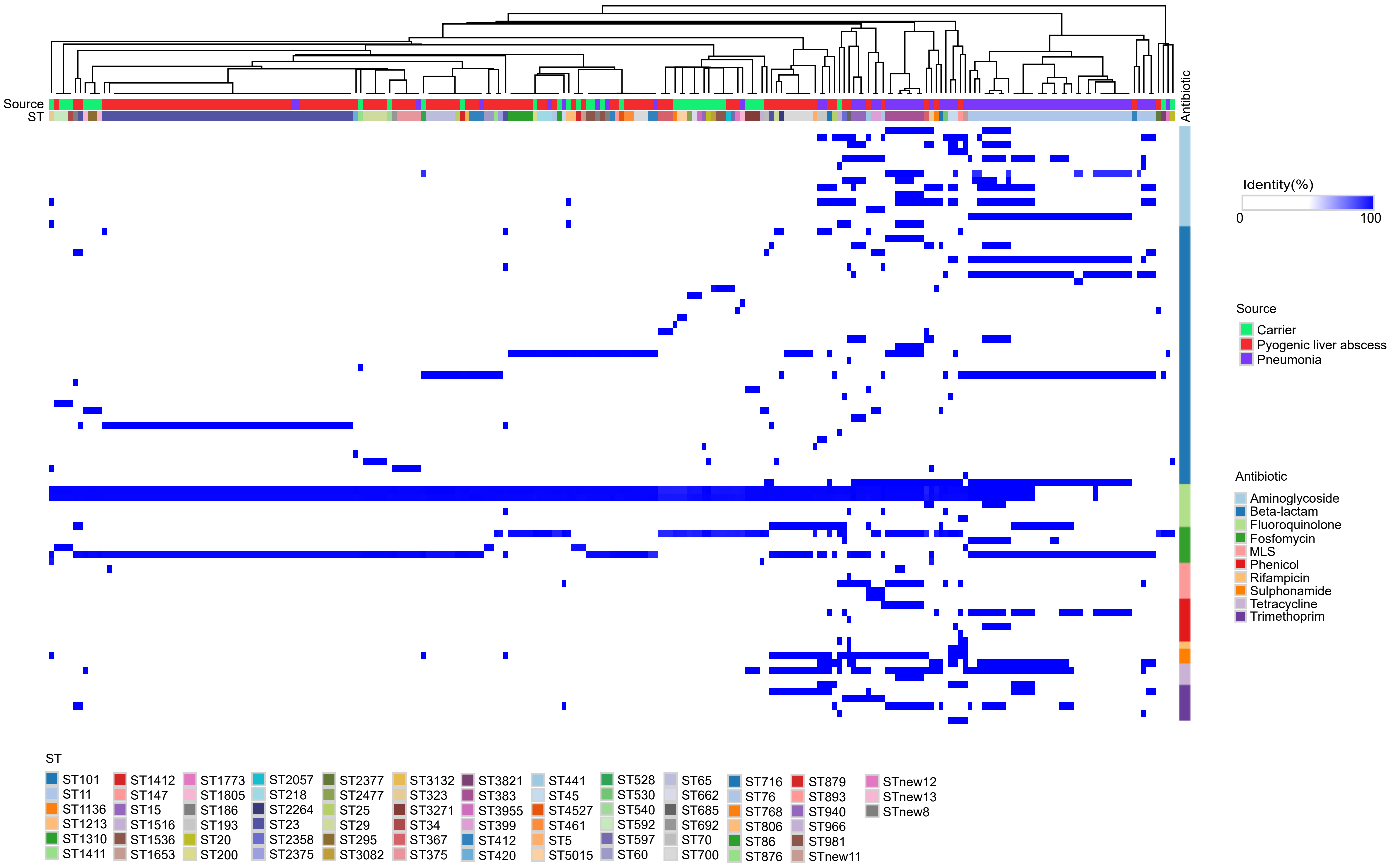

Source
ST
Antibiotic
Identity(%)
0
100
Source
Carrier
Pyogenic liver abscess
Pneumonia
Antibiotic
Aminoglycoside
Beta-lactam
Fluoroquinolone
Fosfomycin
MLS
Phenicol
Rifampicin
Sulphonamide
Tetracycline
Trimethoprim
ST
ST101
ST11
ST1136
ST1213
ST1310
ST1411
ST1412
ST147
ST15
ST1516
ST1536
ST1653
ST1773
ST1805
ST186
ST193
ST20
ST200
ST2057
ST218
ST2264
ST23
ST2358
ST2375
ST2377
ST2477
ST25
ST29
ST295
ST3082
ST3132
ST323
ST3271
ST34
ST367
ST375
ST3821
ST383
ST3955
ST399
ST412
ST420
ST441
ST45
ST4527
ST461
ST5
ST5015
ST528
ST530
ST540
ST592
ST597
ST60
ST65
ST662
ST685
ST692
ST70
ST700
ST716
ST76
ST768
ST806
ST86
ST876
ST879
ST893
ST940
ST966
ST981
STnew11
STnew12
STnew13
STnew8

Figure S3

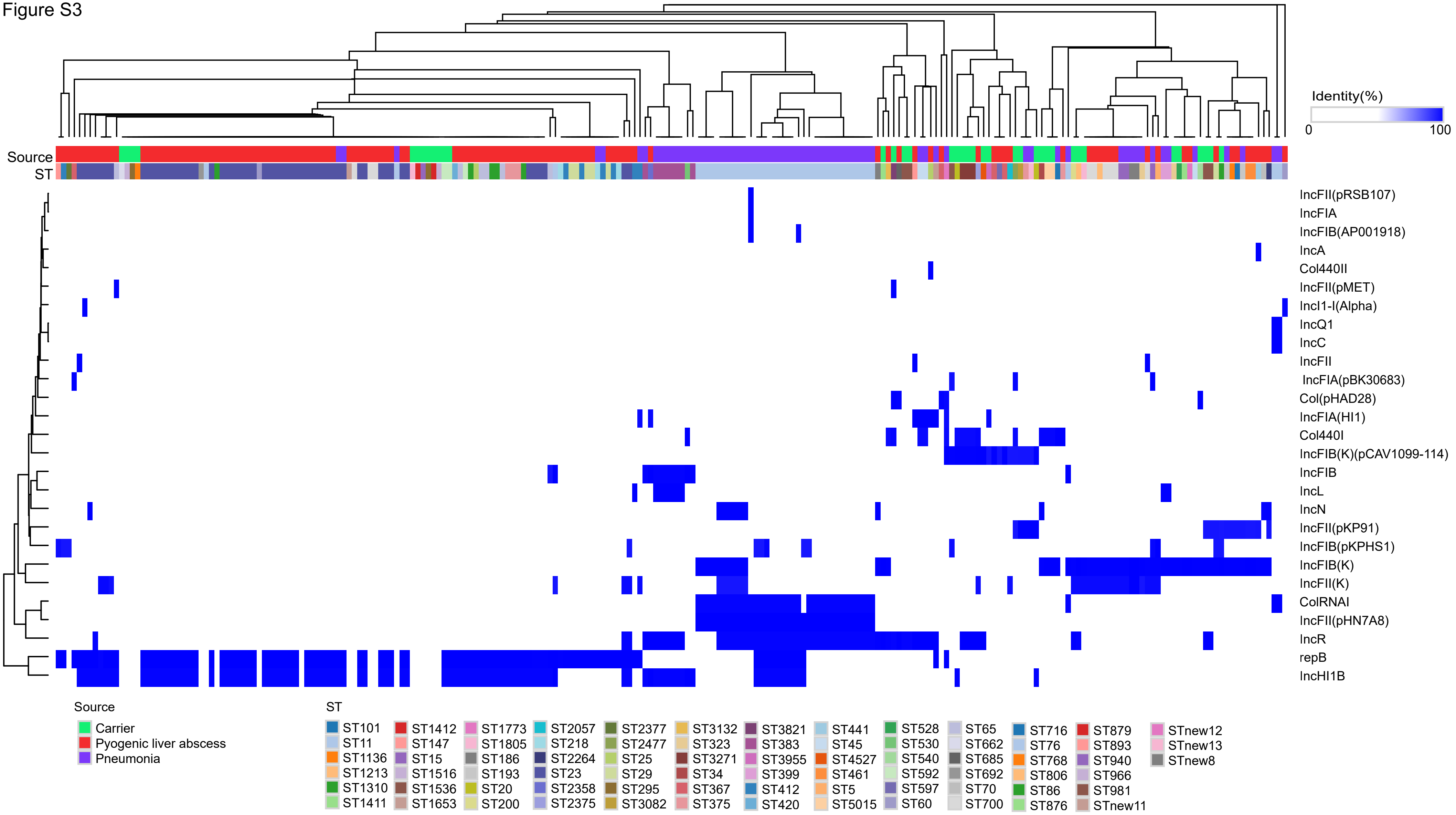
Identity(%)
0
100
Source
ST
IncFII(pRSB107)
IncFIA
IncFIB(AP001918)
IncA
Col440II
IncFII(pMET)
IncI1-I(Alpha)
IncQ1
IncC
IncFII
IncFIA(pBK30683)
Col(pHAD28)
IncFIA(HI1)
Col440I
IncFIB(K)(pCAV1099-114)
IncFIB
IncL
IncN
IncFII(pKP91)
IncFIB(pKPHS1)
IncFIB(K)
IncFII(K)
ColRNAI
IncFII(pHN7A8)
IncR
repB
IncHI1B
Source
Carrier
Pyogenic liver abscess
Pneumonia
ST
ST101
ST11
ST1136
ST1213
ST1310
ST1411
ST1412
ST147
ST15
ST1516
ST1536
ST1653
ST1773
ST1805
ST186
ST193
ST20
ST200
ST2057
ST218
ST2264
ST23
ST2358
ST2375
ST2377
ST2477
ST25
ST29
ST295
ST3082
ST3132
ST323
ST3271
ST34
ST367
ST375
ST3821
ST383
ST3955
ST399
ST412
ST420
ST441
ST45
ST4527
ST461
ST5
ST5015
ST528
ST530
ST540
ST592
ST597
ST60
ST65
ST662
ST685
ST692
ST70
ST700
ST716
ST76
ST768
ST806
ST86
ST876
ST879
ST893
ST940
ST966
ST981
STnew11
STnew12
STnew13
STnew8

Figure S4

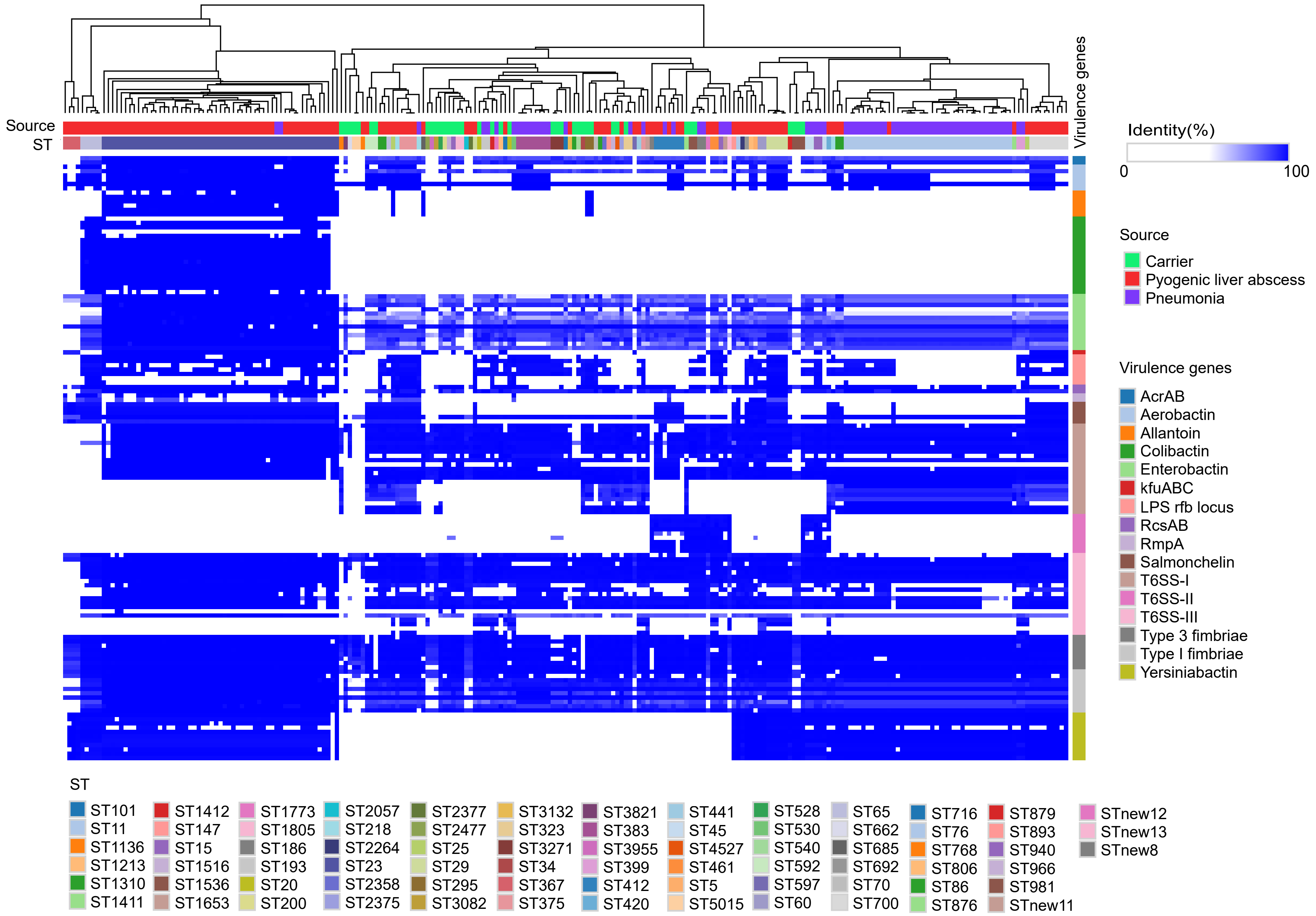

Source
ST
Virulence genes
Identity(%)
0
100
Source
Carrier
Pyogenic liver abscess
Pneumonia
Virulence genes
AcrAB
Aerobactin
Allantoin
Colibactin
Enterobactin
kfuABC
LPS rfb locus
RcsAB
RmpA
Salmonchelin
T6SS-I
T6SS-II
T6SS-III
Type 3 fimbriae
Type I fimbriae
Yersiniabactin
ST
ST101 ST11 ST1136 ST1213 ST1310 ST1411
ST1412 ST147 ST15 ST1516 ST1536 ST1653
ST1773 ST1805 ST186 ST193 ST20 ST200
ST2057 ST218 ST2264 ST23 ST2358 ST2375
ST2377 ST2477 ST25 ST29 ST295 ST3082
ST3132 ST323 ST3271 ST34 ST367 ST375
ST3821 ST383 ST3955 ST399 ST412 ST420
ST441 ST45 ST4527 ST461 ST5 ST5015
ST528 ST530 ST540 ST592 ST597 ST60
ST65 ST662 ST685 ST692 ST70 ST700
ST716 ST76 ST768 ST806 ST86 ST876
ST879 ST893 ST940 ST966 ST981 STnew11
STnew12 STnew13 STnew8
